# Temperature Dependent Growth and Mortality of *Agrotis segetum*

**DOI:** 10.3390/insects10010007

**Published:** 2019-01-06

**Authors:** Peter Esbjerg, Lene Sigsgaard

**Affiliations:** Department of Plant and Environmental Sciences, University of Copenhagen, Thorvaldsensvej 40, DK-1871 Frederiksberg C, Denmark; les@plen.ku.dk

**Keywords:** forecasting, decision support, cutworm attacks, population fluctuations, fecundity, humidity

## Abstract

From 1905 to present, cutworm outbreaks have caused substantial yield losses in North Western (NW) Europe. Early authors pointed to dry summers as the trigger; around 1980, the explanation was improved via modelling of historical data. The number of precipitation days and the July temperature proved to be important, and in experiments, moist soil caused considerable mortality. This information was used in preliminary forecasting with pheromone trap catches as biofix for estimations of occurrence and survival. As more precise information on temperature effects on growth and survival was needed, we performed experiments on growth and mortality effects on egg, all larval instars and pupae. We found clear positive relations between temperatures below 35 °C and development rates of eggs, all larval instars and pupae. Mortality was also affected, and low temperature caused pronounced mortality of young larvae. The severe mortality under cold, moist conditions versus high survival under warm, dry conditions may explain both the lack of relation between captures and injuries and the pronounced fluctuations of cutworm attacks in NW Europe reported from 1905 to present. These variations are likely to increase with the climate change and suggest a reanalysis of data on trap capture and injuries to improve decision support and sustainability in Integrated Pest Management.

## 1. Introduction

The Turnip moth (*Agrotis segetum* Schiff.) is an agricultural insect pest which for more than 100 years has attracted attention in North Western (NW) Europe because of outbreaks in certain years of devastating densities of its larvae and cutworms, causing severe losses in root crops: Denmark 1905 [1], Germany 1917 [2], Estonia 1930 [3], Denmark 1917, 1934 [4], Denmark 1959 [5], Denmark 1975 and 1976 [6,7], Germany 1976 [8,9] Poland 1975 and 1976 [10], and UK 1976 [11]. Early authors such as [4] pointed to exceptional weather conditions during summer as the major cause of the outbreaks and, with reference to the outbreaks in Denmark 1905, 1917 and 1934, dry conditions in May and June were suggested as the underlying factor [4]. An improved background for explaining the outbreaks was produced by graphical estimation of fluctuations in annual attack levels over 70 years [6] on the basis of local attack levels reported by agricultural advisors to governmental authorities. A more detailed graphical analysis of these fluctuations as related to climate showed that excess precipitation in May + June + July prevented major cutworm attacks [12]. This explanation was further elaborated through linear regression modelling, which demonstrated a strong negative correlation between the number of precipitation days in June and July and subsequent attack level [13]. In addition, a strong positive correlation was found between July temperature and the subsequent attack level [13]. The importance of precipitation days subsequently inspired experiments on soil moisture, which revealed moist soil as a significant mortality factor for the first and second larval instars of *A. segetum* [14,15]. This information was an important component of an emerging Danish cutworm forecasting system which used pheromone trap catches as a biofix followed by a simple evaluation of subsequent survival conditions for *A. segetum* larvae derived from semi-field experiments [15]. In this forecasting and later versions, the focus was on eggs and the first three larval instars because control of later instars is inefficient. A remaining gap was to understand the observed strong positive correlation between July temperature and subsequent cutworm attack level [13]. We postulate that this correlation might include two underlying elements: (1) that high temperature increases the growth rate and thus shortens exposure to risk posed by soil moisture for larval instars one (L1) and two (L2), and (2) that larval mortality depends on temperature.

As knowledge of both development rates and mortality as functions of temperature are vital parts of insect pest forecasting, pilot experiments on temperature dependent development rate and mortality of *A. segetum* larvae were performed and the main results were soon transferred into a practical decision support system with the focus on eggs and the first three larval instars [16]. The reliability of the pilot results has been an ending question and also the fact that the preliminary results on growth appeared to indicate differences from similar German parameters on development of *A. segetum* [17,18]. Furthermore, the need for a more precise knowledge of the influence of temperature on growth rate of larvae for forecasting purposes was obvious in the light of the recent results concerning phenology change of *A. segetum* due to climate change effects [19,20]. In this context, better information about temperature-related egg development, and possible egg mortality because of humidity is desirable. We regard egg mortality at low temperature as negligible due to experience with storing *A. segetum* eggs in fridges at 4–8 °C, over periods of up to six weeks [21], but egg mortality due to humid conditions is, as yet, an open question. The eggs are preferably laid on the surface or in between the upper particles of dry soil, but a large proportion of the eggs are laid on lower parts of vertical objects such as plant stems if the soil is moist [22]. The egg structure [23] may indicate a good protection against moisture changes, but high egg mortality due to moist conditions is mentioned by several authors [4,18]. One study [24], observed that eggs exposed to humid air suffered fungal attack and death whilst those exposed to less humid conditions did not, and was later quoted by several authors [4,18]. We have experienced during more than 10 years of rearing *A. segetum*, that under normal conditions (18–22 °C, RH: 60–85%) egg mortality is close to absent, but in earlier work [22], eggs placed on moist material in closed and thereby humid containers were risk prone to fungal overgrowth. We would like to have a minimum of experimental evidence on extreme humidity and moisture effects.

In addition to effects of the above factors, we would also like to know effects of temperature on pupae, particularly females, in order to search for an optimal temperature interval with effect on population build-up and subsequent effect of outbreaks.

We report here the results of four series of experiments, which relate to the numbered hypotheses below:

(1) Development time (and growth rate), mortality, pupal weight and egg number are temperature dependent;
(2) Each larval instar will have specific temperature development thresholds and degree-day (DD) requirements; (The Degree Day concept expresses a combined thermal and time requirement (a heat sum) which is necessary for a particular development. (DD’s permit calculation of occurrence time of insects, and prediction of distributional changes due to climate change).
(3) Egg development is temperature dependent;
(4) Egg mortality is not related to soil moisture and air humidity.

## 2. Materials and Methods

With reference to the above listed hypotheses (1), (2), (3) and (4) this section describes details of each of four experimental series carrying the number of the corresponding hypothesis. Insects for Series 2 were wild, while all insects used for Series 1, 3 and 4 were collected from a culture of *A. segetum* called *As*-culture in the following. The *As*-culture was based on larvae collected during late summer on Danish agricultural sites spread across the country. This culture was at least partly renewed every year and experimental insects were at most from the fifth generation since renewal, and the newest were wild ones. The culture was maintained at 20 °C, LD 16:8 photoperiod and larvae were fed an artificial diet [25].

Series 1. For each replication, neonates were obtained from eggs laid on vertically hanging gauze by 10–15 females, mated by a surplus of males, up to twice as many as females, in a mating and oviposition cage (transparent plastic 20 cm × 30 cm, height 15 cm, 50 cm^2^ ventilation through 2 mm × 2 mm nylon mesh; 2% sugar solution in water as food). Larvae were kept individually in cups with 0.5 cm^3^ of the artificial diet and transferred to fresh cups with new artificial diet when it was necessary to replenish the food. The frequency of this varied with temperature; weekly for specimens at 12 °C and 1–2 times weekly for larvae at other temperatures up to 25 °C and three times a week for larvae at 28, 30 and 35 °C. Series 1 included the following temperatures and larvae (at start): 12 °C, 600 larvae in three replicates of 200; 15 °C, 150 larvae in three replicates of 50; 17 °C, 575 larvae in five replicates of 50, 100 × 2, 125, 200; 20 °C: 325 larvae in five replicates of 50 × 4, 125; 25 °C, three replicates of 50; 28 °C, 750 larvae in six replicates of 50, 100 × 2, 125 × 4; 33 °C: 375 larvae in three replicates of 125; 35 °C: 125 larvae in one replicate.

Checks for instar changes (cast skins and head capsules) and mortality were carried out at 1–2 day intervals depending on age and temperature. The first endpoint was pupation and moth emergence was the final endpoint. 1–2 days after pupation, when the pupae were light brown, each one was sexed [26] and weighed. The time of emergence was recorded and a total of 210 females were collected across temperatures for egg counting by dissection four days after emergence. These females were kept in transparent plastic containers (h = 10 cm, d = 7 cm) with 2% sugar solution provided on a wick, as their food and water supply. The containers were ventilated through a 2 cm × 2 cm opening in the lid covered by filter paper. When dissected, only fully developed eggs in the ovarioles were counted.

In accordance with the DD-concept [27] the rate of development was calculated as the reciprocal value of the number of days to fulfill development at each particular temperature, and subsequently the linear relationship between temperature and the rate of development was calculated by linear regression on data weighted in accordance with numbers of insects per temperature using Proc GLM [28]. The base temperature T_b_ [27] or lower threshold is the intercept on the temperature axis and the reciprocal of the slope of the regression is the thermal constant (DD), which expresses the physiological time requirement for all larval development [27]. Proportion larval mortality through all instars was modelled as M_larvae_ = a + bT + cT^2^. For pupae the same procedure as for larvae was performed to model the development rate, the base temperature and the amount of DD’s required for development from pupation to emergence of a moth. A first full model on all larvae until pupation included sex, but the effect of sex was not significant (Pr > F = 0.7397, F = 0.12). Therefore, sex was omitted, and a second model was run (Proc GLM, [28]) in two versions. One used all temperatures, while the other omitted 35 °C, which appeared to fall outside the interval which can justify a linear regression [27]. The influence of temperature on pupal weight was analyzed by linear regression on weighted data, and the effect of temperature on egg numbers per unit pupal weight was analyzed using a polynomial model:

(1) $$\frac{\log_{10}(\mathrm{Egg})}{P_{\mathrm{weight}}}=a+\mathrm{bT}+{\mathrm{cT}}^{2}$$

where Egg is the number of eggs laid by a female, P_weight_ is pupal weight and T is temperature in degrees Celsius. Finally, the effect of temperature on pupal mortality was analyzed using a polynomial model:

(2) $$R_{\mathrm{dead}}=a+\mathrm{bT}+{\mathrm{cT}}^{2}$$

where R_dead_ is proportion dead, and T is temperature in degrees Celsius.

Series 2 used larvae from 24 hourly egg batches (each batch is the oviposition result of one female in 24 h) laid by 36 wild *A. segetum* females, which were collected as full-grown larvae at eight locations across (east–west) Denmark. Mating and oviposition took place in cylindrical plastic containers (h = 10 cm, d = 7 cm) with a 2% sugar solution from a wick as food and water supply, and a 2 cm × 9 cm hanging strip of gauze for oviposition. Each container held one female and two males. Eggs were removed every day and batches of more than 60 eggs were placed for hatching at 20 °C (incubators as Series 1: L 16:D 8). From each egg batch five sub-batches with 10–25 neonates each were collected 1–12 h after hatching, and the five sub-batches were placed at 15, 17, 20 and 25 °C, respectively (incubators as Series 1, L 16:D 8). In total, 123 neonates started at 15 °C, 145 at 17 °C, 118 at 20 °C, 106 at 25 °C. Each neonate was placed in a semi-transparent 25 mL plastic cup (h = 4 cm, bottom d = 2.5 cm) closed with a plastic lid, and with 0.5 cm^3^ artificial diet. Each individual was checked daily for moulting and at five-day intervals clean cups and fresh food were supplied. L larvae were monitored until molting into L4.

The temperature dependent growth rates of the L1, L2 and L3 were modelled by linear regression (Proc GLM [28]) on weighted data. The temperature dependent mortality of L1 was described by a linear regression of arcsine of proportion dead as a function of temperature (Proc GLM [28]). A similar modelling of L2 was not possible because of extraordinaire mortality at 17 °C.

Series 3 investigated the development rate of eggs used, with batches collected daily from 36 females collected from the *As*-culture. Eggs were laid on gauze strips by females placed in containers as described above (Series 2) and only daily batches with enough eggs to yield five gauze pieces with at least 15 eggs per piece were used. These five batches were placed at 15, 17, 20, 25 and 28 °C, respectively (incubators as Series 1, L:D = 16:8), in the same type of 25 mL cups as used in Series 2. Hatching was recorded daily, but to ensure ready food for the neonates at egg hatching 0.5 cm^3^ of artificial diet was placed in each cup. An accidental fungal infection of some of the diet caused severe overgrowing with a mould toxic to the neonates and these cups had to be discarded. Therefore the final number of batches tested per temperature varied: n, 15 °C = 9 (mean = 52.7, SD = 23.3), n, 17 °C = 18 (mean = 35.7, SD = 16.1), n, 20 °C = 28 (mean = 31.6, SD = 11.9), n, 25 °C = 19 (mean = 38.2, SD = 23.6), n, 28 °C = 18 (mean = 34.1, SD = 13.1). The temperature dependency of the egg development rate was calculated by linear regression (Proc GLM [28]) on all data.

Series 4 studies comprised one part on the possible influence of relative air humidity (RH), using the outermost points of dry versus very humid (almost saturated) air and another part on the possible influence on soil moisture (SoilM) on eggs placed directly on soil, using very dry versus very moist soil. The RH mimics the conditions for *A. segetum* eggs laid above ground on vertical objects, while the SoilM mimics the condition for eggs laid on (or in) the soil [22]. For both RH and SoilM the temperature and light conditions were as for the *A. segetum* culture (Series 1).

For the RH experiment eggs were placed, in the same type of 25 mL cups as used in Series 2, with a strip of gauze with eggs on hanging across the containers’ width, the ends of the gauze being held in place by the lid to avoid direct contact with the four layers of soaking wet filter paper at the bottom of the humid air cups. According to recordings of RH with Georg Jensen^®^ mini hygrometers (inside cups) the dry air cups had RH values between 30% and 45% while humid air cups ranged from 99–100% RH, and often droplets were observed on the lids. For the RH, egg batches from 32 females, collected from the *As*-culture, were used. Each egg batch represented eggs laid by one female within 24 h, and these eggs were separated into two approximately equal parts by cutting the gauze with eggs on under a stereo magnifier. Ten eggs per half was the minimum accepted, and each half was placed in a humid air cup and a dry air cup, respectively. In total 46 egg batches (6813 eggs) from the 32 females were used in this experiment.

For the SoilM experiment, each quarter of an egg batch was placed on a 0.5 cm soil layer at the bottom of a 25 mL cup (as above) and the four cups per batch included four different soil conditions: (1) sterile, dry; (2) sterile moist; (3) non-sterile dry; and (4) non-sterile moist. The purpose of using sterile versus non-sterile soil was to ensure the potential disclosure of microbial effects in case of high mortality of eggs on non-sterile moist soil. The test soil originated from a sandy field (51.1% fine sand and 31.1% coarse sand [15]) known for frequent cutworm attacks, and this soil has been described with regards to its water holding capacity [15] and it had been previously used for oviposition experiments [22]. Sterile soil was obtained by heating soil from the same field at 120 °C for 24 h. For the part with moist soil, a high moisture content of this very dry soil was achieved by wetting the soil of each cup initially with 2 mL of water and then with a further 1 mL of water daily during the experiment. Immediately after wetting, the moist soil appeared very muddy and contained 29–32% water by weight. This percentage dropped to 18–22 prior to the next wetting. The dry, non-watered soil (for the dry part), which contained 10–12% of water, appeared dusty and had the same low water content throughout the experiment. The water percentages were determined by weighing 10 samples of soil per desired measure, then drying for 24 h at 125 °C, and finally reweighing the soil samples. For the SoilM, 28 egg batches (a total of 5376 eggs) from 21 females were used. The effect of dry and humid air was tested with a binomial model for proportion dead and with batch as a random variable in a generalised linear model (Proc GLIMMIX [28]). The effect of dry versus moist soil was tested with a Wilcoxon Two-Sample Test [28], first with the variable proportion dead (in batches) classified by the variable sterile and next with the proportion dead classified by the variable dry.

## 3. Results

**Figure 1:**
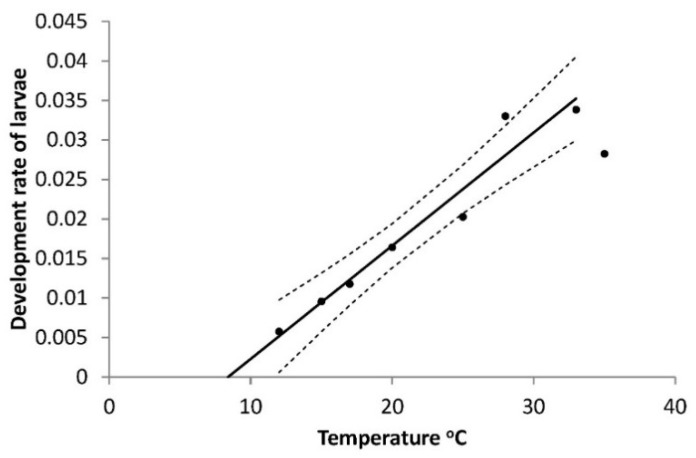
Temperature dependent development of all larval instars of *Agrotis segetum* at eight different temperatures; R_larva_ = 0.0121 + 0.00144T (°C), R^2^ = 0.906, *p* = 0.0001. The highest temperature, 35 °C, is not included in the calculated curve as this temperature appears to fall outsides the interval, which can justify straight line. The dashed lines above and below represent the upper and lower 95% confidence bands.

The larval development time (Series 1) was clearly dependent on temperature (T) as can be described by the equation on the development rate (R_larva_) for all larval instars as a function of temperature, at all temperatures except 35 °C, as this temperature appeared to be outside the interval which can justify a straight line (Figure 1).

**Figure 2:**
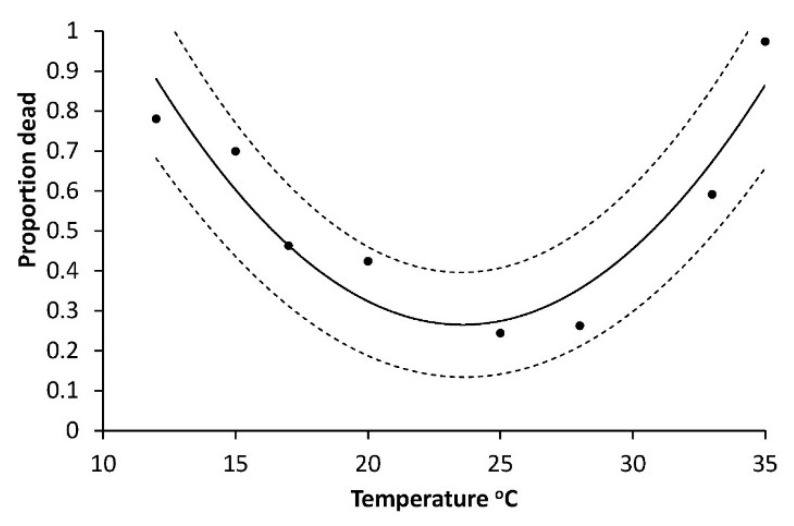
Proportion mortality through of all larval instars (from neonate until pupation) of *Agrotis segetum* at eight different temperatures. Proportion dead = 2.81957 − 0.2167T + 0.0046T^2^, R^2^ = 0.874, F = 17.36, *p* = 0.0056. The dashed lines above and below represent the upper and lower 95% confidence bands.

The result is R_larva_ = 0.0121 + 0.00144T (°C), R^2^ = 0.906, *p* = 0.0001, the base temperature (T_0_) is 8.64 °C and the heat sum required over this temperature was 714 DD. As also mentioned in Materials and Methods, there was no significant difference between the two sexes (F = 0.13, *p* = 0.7227). Mortalities of larvae (all instars, Series 1) also depended on temperature, being lowest around 25 °C and highest at the end points of 12 °C and 35 °C: R^2^ = 0.8741; F = 17.36; *p* = 0.0056 (Figure 2).

**Figure 3:**
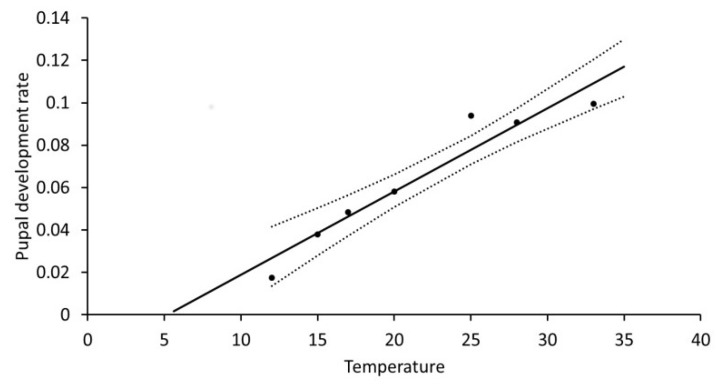
Temperature dependent development of *Agrotis segetum* pupae) at seven different temperatures; R_pupa_ = 0.1886 + 0.0039T (°C), R^2^ = 0.947, *p* = 0.0002. Both sexes are involved, as no statistical difference was found between the two sexes. The dashed lines above and below represent the upper and lower 95% confidence bands.

As it was difficult to categorize a tiny larva as moribund or dead, all moribund larvae were recorded as dead first instars, further supported by lack of head capsules from moulting, and only the precise time of death cannot be given. In contrast, the major mortality at 35 °C was easily observed to take place in the pre-pupal phase. The pupal development rate (R_pupa_) also depended strongly on temperature: R_pupa_ = 0.02252 + 0.0040T (°C), R^2^ = 0.925, F = 62.02, *p* = 0.0005 (Figure 3). This rate was without influence of sex as there was no difference between the two sexes (F = 0.12, *p* = 0.7397). The base temperature (T_0_) for pupal development was 5.61 °C and 250 DD over this temperature were required.

**Figure 4:**
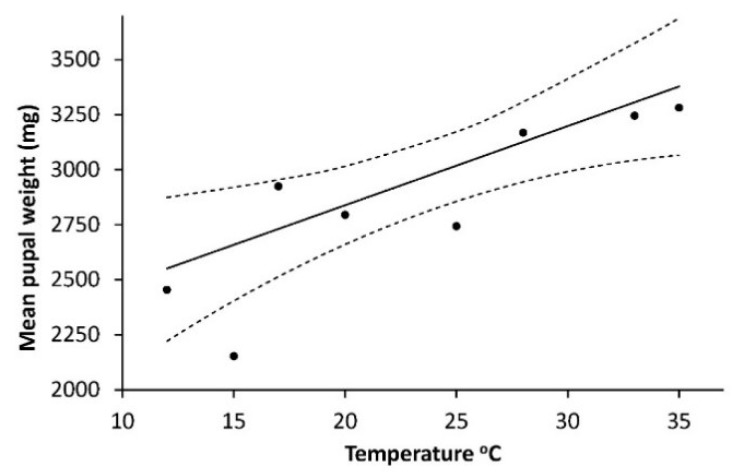
Pupal weight of *Agrotis segetum* as function of temperature. Pupal weight (mg) = 2119.423 + 35.956 T. R^2^ = 0.688, F = 13.25, *p* = 0.011. Both sexes are involved, as no statistical difference was found between the two sexes. Therefore, sex was removed from the final model. The dashed lines above and below represent the upper and lower 95% confidence bands.

As shown on Figure 4, the weight of pupae (W_pupae_) (mg) had a positive linear relationship to temperature: W_pupa_ = 2119.423 + 35.956T, R^2^ = 0.688, F = 13.25, *p* = 0.0011.

**Figure 5:**
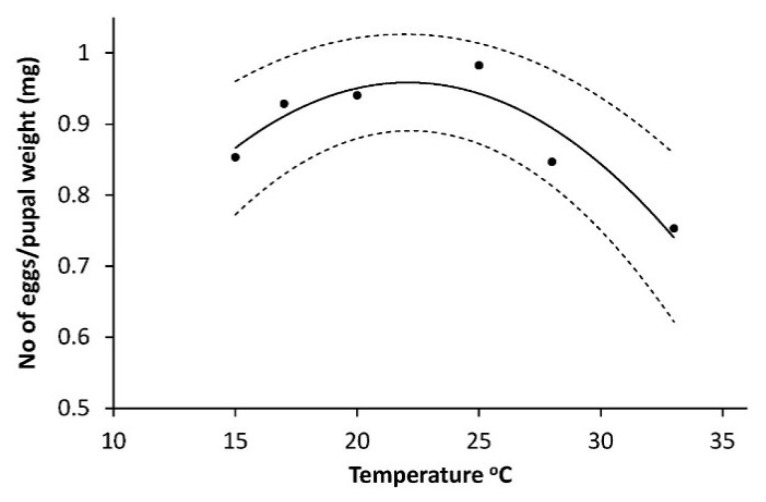
Temperature dependent egg number per mg pupal weight for *Agrotis segetum* pupae at eight different constant temperatures calculated as log_10_ (N_egg_)/gram pupal weight = 0.0630077616 + 0.08108075T − 0.001835228T^2^, R^2^ = 0.866, F_2,3_ = 11.4, *p* = 0.049. The dashed lines above and below represent the upper and lower 95% confidence bands.

Influence of sex had no effect (F = 0.07, *p* = 0.7894) on pupal weight and was removed from the final model. The logarithm of number of eggs (N_egg_) per mg pupae (log_10_(N_egg_)/P_weight_) had a curve linear dependency on temperature with a maximum between 20 °C and 25 °C: (log_10_(N_egg_)/P_weight_ = 0.0630077616 + 0.08108075T − 0.001835228T^2^ (R^2^ = 0.866, F_2,3_ = 11.4, *p* = 0.049) shown on Figure 5.

**Figure 6:**
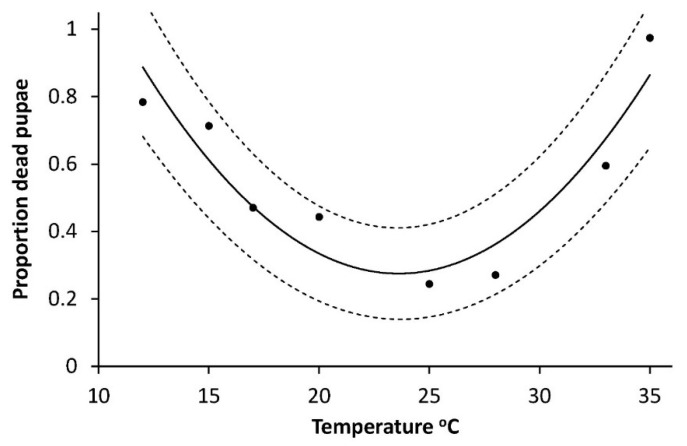
Temperature dependent mortality of *Agrotis segetum* pupae at eight different constant temperatures was modelled as proportion dead pupae = a + bT +cT^2^: Proportion dead pupae = 3.3658 − 0.2904T + 0.0062T^2^; R^2^ = 0.817, F = 11.28, *p* = 0.0140. The dashed lines above and below represent the upper and lower 95% confidence bands.

Also, the mortality of pupae had a curve linear dependency of temperature (Figure 6) but with the minimum between 20 and 25 °C (modelled as M_pupa_ = a + bT + cT^2,^ (M_pupa_ = 3.3658 − 0.2904 × 0.0062, R^2^ = 0.817, F = 11.28, *p* = 0.0140). The highest mortalities occurred at the lowest and the highest temperatures, respectively (Figure 6).

From Series 2, the age specific development rates of the first three larval instars were calculated (Table 1) together with significance levels of parameters and the calculated T_0_ and the DD amount required for each of these instars. The rate of development was significantly dependent on the temperature for all three larval instars, and for the first two the intercept was statistically significant, though this was not the case for L3 (Table 1).

**Figure 7:**
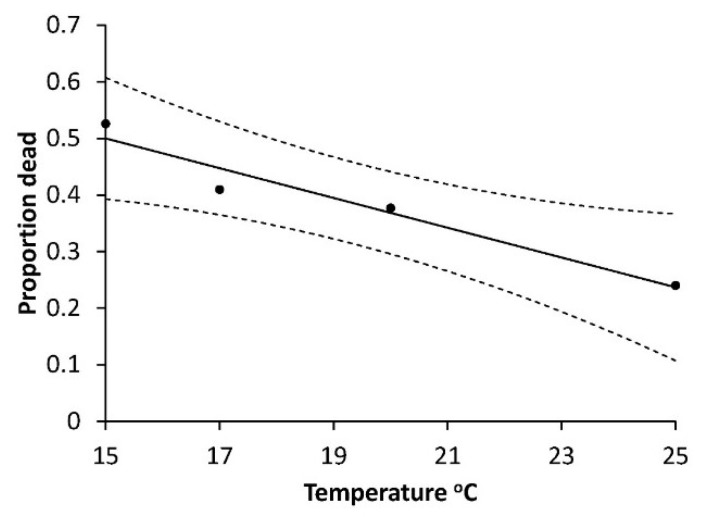
Model description of temperature dependent mortality of first instar larvae of *Agrotis segetum* at four constant temperatures with arcsine to proportion mortality as function of temperature. Arcsine proportion mortality = 0.8946 − 0.0263T. R^2^ = 0.947, F = 35.96, *p* = 0.0267. The dashed lines above and below represent the upper and lower 95% confidence bands.

The mortalities of young larvae at low temperatures (12 and 15 °C) were high in Series 1) and further we saw that the mortality of L1 and L2 decreased with rising temperatures (12 and 15 °C) in a consistent manner which was described for L1 in series 2 (Figure 7) by the negative correlation between mortality described by arcsine proportion dead and temperature (R^2^ = 0.947; F = 35.96, *p* < 0.0267).

In addition, the calculated L1 base temperature (T_0_) of 12.60 °C and the observations of many moribund larvae at 12 °C in Series 1 indicated a high or very high mortality at that temperature. Therefore, we suggest that the major part of the 93% mortality observed at 12 °C happened mainly during L1, with some possibly during L2.

**Figure 8:**
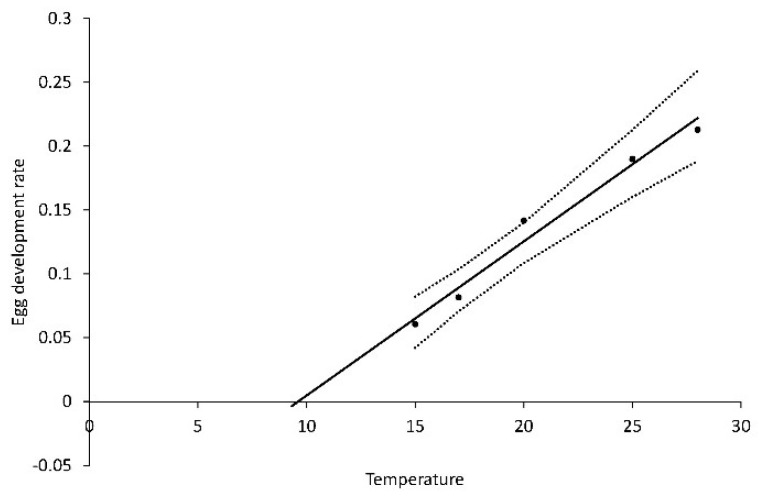
Temperature dependent development of eggs of *Agrotis segetum* at five temperatures; R_leggs_ = 0.1130 + 0.00121T (°C), R^2^ = 0.854, *p* = 0.0001. The dashed lines above and below represent the upper and lower 95% confidence bands.

From series 3, the development rate of eggs (R_eggs_) was modeled as: R_eggs_ = 0.1130 + 0.0121T, R^2^ = 0.854, *p* < 0.0001. (Figure 8). The heat requirement was 83 DD over a base temperature (T_0_) of 9.3 °C.

From series 4, the part on air humidity influence on eggs, showed a mortality of 14.4% for eggs exposed to very dry air and a corresponding figure of 10.0% for eggs exposed to humid air. The mortality across the two treatments was 12.2%. The difference in mortality between the two treatments was significant (F = 48.02, *p* < 0.0001). The part on soil moisture did not reveal significant differences (Wilcoxon test), neither between eggs on sterile versus non-sterile soil, nor between dry versus moist soil. In the latter case, the mortality on dry soil was 5.9%, and on moist soil the similar mortality of eggs was 2.9%. The mortality across all treatments and including batches with zero mortality in all treatments was 4.4%.

## 4. Discussion

Through our results, we have obtained important answers to the two introductory questions regarding the strong influence of the July temperature found by [13]. Thus, high and low temperature will critically affect survival as well as development of L1 and L2 in particular. This should also be seen in the light of the interaction between soil moisture and upper soil temperature, as ongoing soil moisture will cool the upper soil, while high temperature, particularly due to sunshine, will dry out the upper soil. That interaction will be further discussed below together with egg mortality, which we have found, is basically low, and mortality of young larvae, which may vary strongly. Overall, we have elucidated important aspects of temperature influence on the juvenile *A. segetum* life stages. This information can add to a better understanding of population fluctuations and, in particular, form an improved background for forecasting and decision-making, which must take into account that the very last control option is at the second instar for organic growers, using irrigation as control measure, and for conventional growers, using chemical control, at the early fourth larval instar.

As hypothesized (3) the development of eggs depends on temperature and can be described by a linear regression with development rate as a function of temperature yielding a base temperature of 9.3 °C and a thermal constant of 83 DD following the methodology described by [27]. Our results also confirm that in terms of days needed for development of *A. segetum* eggs at three selected temperatures there is some difference from previous German results [17], which were quoted by [18], and which are the only data available for comparison. The German *A. segetum* eggs needed four days at 26.7 °C, while Danish *A. segetum* eggs will develop in 4.8 days at that temperature, which is approximately one day more than German eggs from 300–500 km south of Denmark. However, the German eggs developed in 12 days at 18 °C [17] in contrast to 9.5 days needed at 18 °C for Danish eggs in this study. There could be some difference between the strains studied explaining the different results, but the difference may also be an artifact of available equipment at the time of the earlier study. In any case, it is not a problematic difference in a practical setting.

The present results on Danish eggs show that the preliminary results [16] are only slightly different from the present, for example in terms of an old base temperature of 10 °C while the new is 9.3 °C. At a crucially high average topsoil temperature of 25 °C, this difference only causes a need for 0.2 days more for egg development—essentially nothing in practice. More interesting, maybe, is the calculation of how slowly eggs develop under colder conditions. Thus, 14.6 days are needed at 15 °C, which is a common average soil temperature [29], and at 13 °C, which may also occur, 22.4 days are required for egg development. The time difference between warm and cold conditions is very important for timing in forecasting.

The mortality of eggs at different temperatures can almost be ignored. Our experience from keeping eggs in fridges and the laboratory indicate that temperatures between 4–6 °C have a negligible, if any effect on egg mortality and similarly room conditions of 16–2 °C. Further, our results do not fully support the hypotheses (4) that extreme air humidity and soil moisture conditions will not have a mortality effect on eggs. The results show that there are in fact limited mortality effects in very dry air (14%) and very humid air (10% mortality). Despite the significantly higher mortality in dry air, in practice it would be of no importance, as the very low relative humidity we have used is not likely to occur during the Danish growing season, except for very short time intervals around noon. The mortality caused by very dry and very moist soil, respectively, was very low (3–6%), and there was no significant difference. Even though we cannot completely reject egg mortality at intermediate humidity and soil moisture conditions, we believe effects stronger than at the humidity and moisture extremes are unlikely. Therefore, we anticipate that the total mortality of eggs will remain within 12%, and hence the egg phase mortality is neither likely to contribute significantly to the background for forecasting, nor to add to the explanation of major population fluctuations.

In contrast to the egg phase, the early larval phase has a known risk of high mortality due to moist soil [14,15]. Low temperature may also cause high mortality particularly of L1 and L2, and, furthermore, these instars develop slowly at low temperature of 12 and 15 °C and much faster at higher temperatures (25 and 28 °C) as hypothesized. L1 has a calculated requirement of 51 DD over 12.6 °C, and L2 requires 41 DD over 11.7 °C (Table 1). The relatively high base temperature requirements of the first two instars resembles what is known from some other Noctuids as for example *Mamestra brassicae* (L) [30] and *Egira curialis* (Grote) [31]. The development rate found for L1 at moderately high temperature (20–22 °C) does not really differ from the earlier German results [18]. At 22 °C, German L1 *A. segetum* developed in 5.5 days while Danish ones needed 5.4 days. Further, our rate of development for L2 at 25.2 °C is nearly the same as the older German results [17]. We can conclude that for the whole L1 + L2 period there is not a forecasting relevant difference between Danish and German *A. segetum*. For L3 our results are unfortunately not as solid as for L1 and L2 as the intercept is not significant (Table 1) but if we neglect this and use the calculated base temperature and DD requirement, the outcome at 25.2 °C is 5.1 days, compared to the 5 days of [17]. This is effectively the same and strengthens the case for using these results in IPM programs unless and until experiments with more larvae yield a different outcome. Our results from Series 1 (Figure 1) are shown in accordance with the common tradition of doing so (e.g., [32,33]) but in the light of the contrast between the base temperature for all larval instars (8.6 °C) and those of L1 (12.6 °C) and L2 (11.7 °C) the value of the relation for all larval instars can be questioned and should not be used in IPM programs. More important is the new finding of pronounced mortality effect of low temperature (12 and 15 °C, Figure 2), which was never mentioned by earlier authors [17,18] and it appears to be an overlooked element. The importance of L1 mortality appears from Figure 7 and was also high for L2, and we can conclude that the mortality of young larvae may account for the major part of high total mortality, shown by the 93% mortality at 12 °C (Figure 2).

From monitoring and forecasting results of the HortiAdvice Scandinavia [34], a forecasting which uses preliminary results on development [16], it seems that the major occurrence of L1 and L2 will generally take place in the first half of July. As our present result on these larval instars’ temperature dependent development only differ little from the preliminary results [16], July mortality should be a focus of interest, and from field recordings it is known that even in July (and August), the statistically warmest months in Denmark [35,36,37], the monthly average temperature of grass covered soil may stay at 15 °C [29] and colder periods of one to three weeks may occur. This means that a cold July will cause a considerable or even high mortality, which will further increase if the soil surface is moist for a sufficiently long time [14,38]. In this context, the effect of a much longer development time at low temperature (e.g., 25 + 12 days to pass L1 and L2 at 15 °C) also has to be taken into account as the phase most risk prone to these conditions is prolonged. Consequently, after a cold period the risk of subsequent attacks is strongly reduced, or even eliminated. The opposite situation will be a warm and dry period, long enough to bring the young larvae through to L1 and L2 without any detrimental coldness or soil moisture (e.g., 7–8 sunny, dry days with dry upper soil around 25 °C). Then, a very high survival is likely and the risk of subsequent attacks on crops may be considerable. With this contrast between chill and moist versus warm and dry, the influence of July temperature as found by [13] appears obvious. On this background we conclude, that different combinations of weather within the continuum from warm and dry (fast passing of L1–L2 with low mortality) to cold and humid (slow passing of L1–L2 with high mortality) can explain the very strong variation in late occurrence of damaging cutworms even after comparable levels of preceding trap catches of turnip moths [38]. This is most likely also a major reason for the previously unexplained population variations over the past 70 years [6,13].

Despite the absolutely dominating influence of the L1–L2 fate in terms of mortality/survival, the fate of L3 also depends considerably on soil moisture and is also affected by low temperature [15]. Thus, a long cold and moist July period may almost wipe out cutworms [38], whereas a prolonged dry and warm period can promote such serious cutworm attacks as the extreme ones in 1976 [7,8,9]. The year 1976 was exceptional following an already exceptional summer in 1975 [39]. Furthermore, it appears from light trap catches [40] that the 1976 summer may have enabled two full moth generations sufficiently early that the second also delivered large cutworms. This is in contrast to the phenological trap with a lost second generation of larvae (not reaching maturity for hibernation) as found at present for Danish *A. segetum* [19,20].

With the climate change a two full generation situation is more likely to occur again, and cutworm devastating precipitation is already more common [41]. Interesting for the general understanding of the population dynamics of the species are also our findings that the sex ration is 1:1 and the optimal temperature for producing the highest number of eggs per mg pupae is around 22–23 °C (Figure 5). This might tell that an exceptional summer may not only lead to a higher population but also to a somewhat higher female fecundity the following year. Our finding regarding eggs per pupal weight may also explain why authors from several more southern countries find a higher average egg number per female [18,26] 1983) than in Denmark. It is also noteworthy that the overall picture is that a soil temperature close to 25 °C appears to be an optimum where the development rate is rather high (L1, L2 and L3, Table 1), the mortality is low (Figure 2), and the production of eggs at a maximum (Figure 5). This might further help explaining why attack levels in the most reported “cutworm summer” of 1976 reached devastating levels due to the very long, warm and dry period over a considerable part of NW Europe.

On the basis of the present results it can now be considered whether the emergence of the *A. segetum* moth in early summer can be predicted through modelling of data from the previous year and weather data from the early part of the actual year. This option seems attractive, but there are some major obstacles for doing this. (1) The larvae which approach overwintering are exposed to a quite different day length in the later part of their life, and this will change their growth rate as found in Germany [18]; (2) It is unknown what triggers the termination of overwintering at a soil depth of 3–7 cm [42] and make the larvae move up to 1.5 cm below the surface, where they make a pupation chamber. The move seems to occur at a soil temperature of 10–11 °C [42]; (3) Further, it is not known whether the larvae preparing for pupation after overwintering need a particular thermal sum before pupation, nor do we know whether the pupal DD request differs from what we have found under summer like conditions. We therefore conclude that with the present information, the post winter emergence time of this moth cannot be modelled.

The management implications of our rather precise results, which can largely confirm and add precision to the preliminary results on development used before, are generally important but mostly the new key element is temperature-dependent mortality, which is a crucial factor with the changing climate. Thus, we can apply a good local biofix for calculations by using the trap catch level within a defined time period (a week including two counts) followed by an egg maturation and oviposition period of five days in accordance with findings from southern Sweden [18,43]. At single location level the time-periods for egg development, for presence of L1, L2 and L3, respectively, can all be calculated on the basis of the specific DD requirements found. For each of the larval instar periods, we can make a reasonable assessment of larval survival/mortality conditions based on information about soil moisture and temperature leading to a decision of no control under the present conditions, mechanical control by irrigation against L1 and L2 (mostly used in organic production), later chemical control in conventional production, or a final no-risk of damage message.

These steps have been used in the preliminary Decision Support System [16] but the uncertainty about correct timing and estimation of larval mortality can now be strongly reduced. So far, the Danish Decision Support System from an array of years appears successful both in Denmark and southern Sweden, and most noteworthy is perhaps the mechanical control by irrigation of tiny cutworms in organic production which is highly dependent on proper timing. This represents new progress. With the negligible differences between our results and earlier German results on temperature dependent growth, it might be possible also to use our more comprehensive results in decision making in the northern part of Germany and Poland.

A final aspect of our results is the opening for analysis of non-systematic injury assessments as related to preceding trap captures over 30 years. This may enable evaluation of forecasting and DSS up to now: which yield losses were avoided, and how much the frequency of treatments could be lowered in comparison with a management based on scheduled treatments. Last, but not least, there is now an option for using scattered data from the 1980s up to now on injury levels assessed in fields to establish a possible proper relation between trap catches, with adjustments for subsequent mortality of L1 and L2, and the final crop injury level. This will enable adjustments of a set of control thresholds [16] which may for long have needed an overhaul. Further, it may contribute to building IPM standards in accordance with the EU Directive 128 of 2009 [44], on more sustainable use of pesticides and to ensure sustainability through up-to-date thresholds as recently underlined [44].

## 5. Conclusions

We have elucidated the influence of temperature on growth and survival of eggs, larvae, early instars in particular, and pupae. Through this we have determined thermal requests with emphasis on eggs and early larval instars. Our findings on growth rates do not either differ much from historical and more recent German results, nor from preliminary Danish results, but a background for improved forecasting of attacks has been established. We have also demonstrated that while eggs are rather robust to both various temperature and moisture conditions, this is not the case for larvae. Thus, we have found that low temperatures (12–15 °C) cause high mortality of the first two larval instars. This effect was apparently overlooked before and can interact with the known effect of moist soil. We conclude that the fate of the first two larval instars can explain the until now open question about importance of July temperature for the strength of subsequent attacks of large cutworms. We also regard the fate of these two larval instars as the key to understand both the strong local and annual variation in attacks and the fact that a considerable trap capture of parent moth may not necessarily lead to a cutworm attack. This understanding is of growing importance due to the increasing annual and local variation in temperature and precipitation as a consequence of climate change. With our results as well, decision support to growers as well as the general recognition of population dynamics can be improved. As a final perspective we suggest that our results can enable a more detailed analysis of the relation between trap capture data and root crop injuries in order to improve control thresholds [45] in particular and decision support in general.

## Figures and Tables

**Table 1:** Linear models of development rates for 1st, 2nd and 3rd instar larvae of *Agrotis segetum* at temperatures of 15, 17, 20 and 25 °C, showing a clearly significant effect of temperature and significant intercepts for 1st and 2nd instars, but not for 3rd instar. The second to last column shows the calculated base temperature for each of the three instars and the last column shows the calculated heat requirement in Degree Days.

Instar	R^2^	F	Pr > F	Intercept ± SE	T	Pr > |t|	Temp ± SE	t	Pr > |t|	T_0_ (°C)	DD
1st	0.974	75.70	0.013	−0.249 0.044	−5.69	0.030	0.020 0.002	8.70	0.013	12.6 ^A^	51 ^A^
2nd	0.995	370.44	0.003	−0.287 0.025	−11.64	0.007	0.025 0.001	19.25	0.003	11.7 ^B^	41 ^B^
3rd	0.940	31.61	0.030	−0.068 0.036	−1.88	0.201	0.011 0.002	5.62	0.030	6.5 ^AB^	95 ^AB^

Values in the same vertical column followed by different letters are significantly different.
